# Antagonistic Regulation, Yet Synergistic Defense: Effect of Bergapten and Protease Inhibitor on Development of Cowpea Bruchid *Callosobruchus maculatus*

**DOI:** 10.1371/journal.pone.0041877

**Published:** 2012-08-21

**Authors:** Fengguang Guo, Jiaxin Lei, Yucheng Sun, Yong Hun Chi, Feng Ge, Bhimanagouda S. Patil, Hisashi Koiwa, Rensen Zeng, Keyan Zhu-Salzman

**Affiliations:** 1 Department of Entomology, Texas A&M University, College Station, Texas, United States of America; 2 State Key Laboratory of Integrated Management of Pest and Rodents, Institute of Zoology, Chinese Academy of Sciences, Beijing, People's Republic of China; 3 Department of Horticultural Sciences, Texas A&M University, College Station, Texas, United States of America; 4 Vegetable & Fruit Improvement Center, Texas A&M University, College Station, Texas, United States of America; 5 State Key Laboratory of Conservation and Utilization of Subtropical Agro-Bioresources, South China Agricultural University, Guangzhou, People's Republic of China; U. Kentucky, United States of America

## Abstract

The furanocoumarin compound bergapten is a plant secondary metabolite that has anti-insect function. When incorporated into artificial diet, it retarded cowpea bruchid development, decreased fecundity, and caused mortality at a sufficient dose. cDNA microarray analysis indicated that cowpea bruchid altered expression of 543 midgut genes in response to dietary bergapten. Among these bergapten-regulated genes, 225 have known functions; for instance, those encoding proteins related to nutrient transport and metabolism, development, detoxification, defense and various cellular functions. Such differential gene regulation presumably facilitates the bruchids' countering the negative effect of dietary bergapten. Many genes did not have homology (*E*-value cutoff 10^−6^) with known genes in a BlastX search (206), or had homology only with genes of unknown function (112). Interestingly, when compared with the transcriptomic profile of cowpea bruchids treated with dietary soybean cysteine protease inhibitor N (scN), 195 out of 200 coregulated midgut genes are oppositely regulated by the two compounds. Simultaneous administration of bergapten and scN attenuated magnitude of change in selected oppositely-regulated genes, as well as led to synergistic delay in insect development. Therefore, targeting insect vulnerable sites that may compromise each other's counter-defensive response has the potential to increase the efficacy of the anti-insect molecules.

## Introduction

Plants produce a wide range of secondary metabolites, or allelochemicals, to protect themselves from devastation by animals, insects and pathogens. Furanocoumarins are one group of naturally occurring plant secondary compounds that have shown toxicity against a broad spectrum of animals including herbivorous insects, and affect their feeding behavior [1]–[3]. Bergapten (5-methoxypsoralen) is a linear furanocoumarin isolated from plant families including Apiaceae, Rutaceae, Leguminosae and Solanaceae [4]. When ingested by insects, it decreases larval weight, extends generation time and induces mortality [1], [5]. Bergapten can be photoactivated and is capable of crossing-linking DNA, covalently modifying proteins and lipids, and consequently inhibiting cell replication [6]–[8].

Many herbivorous insects, through the long history of interaction with their hosts, have evolved the ability to protect themselves from a variety of toxic plant secondary compounds [6], [9]–[11]. Our current understanding of insect adaptive mechanisms has been mostly derived from extensive biochemical and molecular studies of major detoxifying enzymes, such as cytochrome P450 monooxygenases (P450s), glutathione-S-transferases (GSTs) and catalases (CATs). P450s, catalyzing the oxidation of xenobiotic substances, are the best studied components of insect detoxification systems. The black swallowtail (*Papilio polyxenes*), for example, specializes in feeding on furanocoumarin-containing plants due to development of allelochemical-metabolizing capacities [12]. *P. polyxenes* P450s, encoded by *CYP6B* genes, play a paramount role in furanocoumarin tolerance. Induction of *CYP6B* homologs from generalist *Papilio* species is also responsible for the metabolism of furanocoumarins [6], [13]. Furanocoumarin metabolites via P450s have been characterized in some species [14]. GSTs also mediate resistance to plant allelochemicals. As phase II metabolic enzymes, GSTs are capable of conjugating reduced glutathione to the electrophilic centers of a wide variety of substrates [10], [15], [16]. Further, CATs are key enzymes of the cellular antioxidant defense system, scavenging hydrogen peroxide to avoid oxidative damage to protein, nucleic acid and lipids [17], [18].

Such plant defense and insect adaptation has also been illustrated in inhibitor-protease interactions. Plant protease inhibitors are able to suppress insect digestive enzymes, leading to a reduction in amino acid assimilation by insects [19], [20]. Attempts to use protease inhibitors in transgenic crops, however, have been largely unsuccessful because insects rapidly adapted to the presence of inhibitors in their diet. Strategies utilized by insects include (i) overproduction of digestive proteases to out-titer the inhibitors in insects [21]–[23]; (ii) increased expression of inhibitor-insensitive protease isoforms [24]–[26]; and (iii) activation of proteases that hydrolyze and thus detoxify plant inhibitors [27]–[29].

We have previously used the cowpea bruchid (*Callosobruchus maculatus*), a coleopteran storage pest, as our model insect to study its adaptive mechanisms against soybean cysteine protease inhibitor (scN). This insect is the most serious post-harvest pest of cowpea (*Vigna unguiculata*), related to common bean and chickpea. It plagues cowpea and other legumes in the storage granary and destroys the harvested grains [30]. Cowpea bruchids lay eggs on the seed surface. Larvae feed and develop inside the seeds, and emerge as adults. Even with a minor infestation at harvest, the high reproductive capacity, short life cycle and continuous generations of the bruchid can lead to complete loss of stored grains in a few months. Traditional breeding to introduce insect resistance into cowpea cultivars has largely failed, mainly because the cowpea gene pool lacks useful resistance genes.

scN, the soybean cysteine protease inhibitor, when incorporated into artificial diet, inhibits digestive enzymes of cowpea bruchids and delays their development. However, this negative impact only occurred during the early developmental stage. When the insects reached the 4^th^ instar prior to pupation, the effect of scN diminished despite their continued feeding on scN-containing diet [31]. Midgut transcriptomic profiling indicated that dietary scN induced large scale gene expression changes. Interestingly, while it strengthened insect digestive ability, their disease resistance and detoxification appeared to be weakened, reflected by down-regulation of genes encoding antibacterial peptides or anti-pathogen proteins and detoxification proteins [32]. Such a trade-off presumably allows insects to most efficiently utilize available resources when they are facing specific challenges. Meanwhile, reduced detoxification could represent a new vulnerability that we can utilize to achieve better pest control through simultaneous targeting insect digestion and detoxification.

To gain new insight into insect adaptation to furanocoumarins, in this study we evaluated the effect of bergapten on growth and development of the cowpea bruchid. We also performed a large-scale midgut transcriptomic analysis in response to dietary bergapten. Contrasting expression patterns when compared with scN-regulated transcript profiling led to a further investigation of the combined effect of bergapten and scN, as well as a study to better understand the molecular mechanism of their synergistic anti-insect activity.

## Materials and Methods

### Artificial seeds and insect feeding treatments

Bergapten (Sigma-Aldrich, St. Louis, MO) was dissolved in acetone at a 1% stock concentration. Cowpea seeds (purchased from HEB grocery store) were briefly soaked in distilled water, followed by removal of their testae. After an overnight air-drying, the decorticated seeds were ground into fine flour. Five 250-mg artificial seeds were made for each treatment. Flour was weighed and mixed with distilled water and bergapten solution to a final concentration of 0, 20, 50, 100, 250, 500, 800 and 1000 ppm, respectively. The proportion of the flour and liquid was such that each 1 g of flour was mixed with 1 mL liquid (combination of water and bergapten solution). An equal amount of acetone, the solvent for bergapten, was incorporated into all treatments, including control seeds. Separately, control seeds without acetone were compared to acetone-only controls, to determine possible acetone effects. The well-mixed flour paste was then injected into a pre-chilled Teflon mold using a 10-mL syringe, and the mold was frozen in liquid nitrogen, followed by freeze drying in a lyophilizer.

For scN treatment, recombinant protein obtained as previously described [31] was dissolved in distilled water at a concentration of 2 mg/mL and incorporated into artificial seeds at 1,000 ppm alone, or in combination of 250 ppm bergapten. All treatments including controls contained equal amounts of acetone. After 24-hr lyophilization, seeds were removed from the mold and equilibrated with atmospheric moisture, prior to coating with 8% gelatin.

To assess the effects of bergapten on developmental time and mortality, cowpea bruchid adults were allowed to lay eggs till each seed had 6 to10 eggs. Infested seeds in clear glass bottles were then placed in a growth chamber (26°C, 45% RH, 16L/8D photoperiod), and the number of hatched eggs and newly emerged adults were recorded daily until 60 days after the last adult emerged. The developmental time was defined as the time period from egg laying to adult emergence. Mortality was calculated from the number of emerged adults and the number of successfully hatched eggs, indicated by change of the egg color, from clear to cream white.

To evaluate dietary bergapten impact on fecundity, cowpea bruchids reared on artificial seeds containing 250 ppm bergapten were produced as above. Newly emerged adults (within one day) from these seeds, four female and two male, were placed in a separate bottle and eggs laid were recorded and removed daily till all insects died. Three replicates were performed. Insects obtained from artificial seeds without bergapten served as the control.

### cDNA Microarray analysis

We utilized our previously constructed microarray platform containing 20,352 randomly picked cDNA clones from a normalized cowpea bruchid midgut cDNA library (for detailed information, see [32]). Microarray slides were processed prior to hybridization by blocking in 1% SDS for 10 min at 25°C, followed by DNA denaturation in boiling water for 2 min, and 95% ethanol treatment at −20°C for 2 min.

Midguts (15) from 4^th^ instar bruchid larvae, treated or control, were collected following Zhu-Salzman et al. [31]. Total RNA was extracted from the dissected midguts using a Trizol-based method (Invitrogen, Carlsbad, CA). Cy3- and Cy5-labeled probes were prepared using the 3DNA Expression Array Detection Kit for Microarray from equal amounts of total RNA (Genisphere, Hatfield, PA), and cohybridized to the microarray (For details, see [32]). For each biological replicate, at least two microarray hybridizations were performed.

The microarray slides were scanned with a Packard Scanarray 5000 four-laser confocal scanner (Packard Bioscience, Billerica, MA), and images were analyzed with the Quantarray program (Packard Bioscience). Output data were further processed using GeneSpring 7.3.1 software (Agilent Technologies, Redwood City, CA). Data from 6 slides (3 technical replications ×2 biological replications) were averaged and normalized using the GeneSpring Lowess algorithm for each treatment condition. Results were then filtered by expression fold change and confidence values. Expression data with mean fold changes of more than 2.0, and significance at *P*≤0.05 (Student *t*-test *P* value, using Benjamini and Hochberg F.D.R. multiple testing correction) were retained. The corresponding clones were subjected to sequencing analyses for gene identification.

### DNA sequencing, BLAST search and KEGG analyses

Plasmids of selected clones based on the above criteria were extracted, and insert sequences were determined. Trimming of the vector sequence and oligo(T) from the raw data as well as contig assembling were performed using Sequencher software (Gene Codes Corporation). All contigs and singletons were annotated by NCBI BlastX searches using the BLAST2GO software suite v.2.5.0 (http://blast2go.de) [33]. The E-value cutoff was set at 10^−6^. Annotated bergapten-responsive genes were determined for their biological processes and molecular functions based on Gene Ontology (GO) terms associated with the BLAST2GO program using the default parameters. To analyze the network of bergapten-responsive genes in bruchid midgut, the annotated genes were mapped to KEGG (Kyoto Encyclopedia of Genes and Genomes) metabolic pathways available in the BLAST2GO software.

### Quantitative RT-PCR

Total RNA was extracted from the dissected midguts of treated and control 4^th^ instar larvae, respectively, using a Trizol-based method (Invitrogen, Carlsbad, CA). cDNA was synthesized using Superscript™ II reverse transcriptase (Invitrogen) and random hexamer primers. Primers for selected genes were designed using Primer Express (Applied Biosystems, Foster City, CA). For each gene, duplicate 10 µL qRT-PCR reactions were performed using SYBR Green Mastermix (BioRad, Hercules, CA) as described previously [34]. PCR amplification of 18S rRNA was performed for normalization between treated and control samples. Amplification specificity was determined by dissociation curve analysis. No-template controls using untranscribed RNA confirmed that no interfering products derived from genomic DNA were present. Gene expression levels of samples in each treatment were compared to the levels of the corresponding untreated samples, which were arbitrarily set at 1. Mean induction/suppression fold of each selected gene was calculated following Chi et al. [34].

### Statistics

Statistical analyses were performed using SPSS 10.0 software (SPSS Inc., Chicago, IL). One-way analysis of variance (ANOVA) was used to analyze the effect of bergapten, alone or in combination with scN, on developmental time of cowpea bruchids. If the variable was significant, Tukey's multiple range test was used for pairwise comparison of the difference between treatments for mean separation (*P*<0.05). Significance of the effect of bergapten on fecundity and effect of acetone on developmental time was determined by independent *t*-test.

## Results

### Bergapten negatively impacts survival, development and fecundity of cowpea bruchids

**Figure 1:**
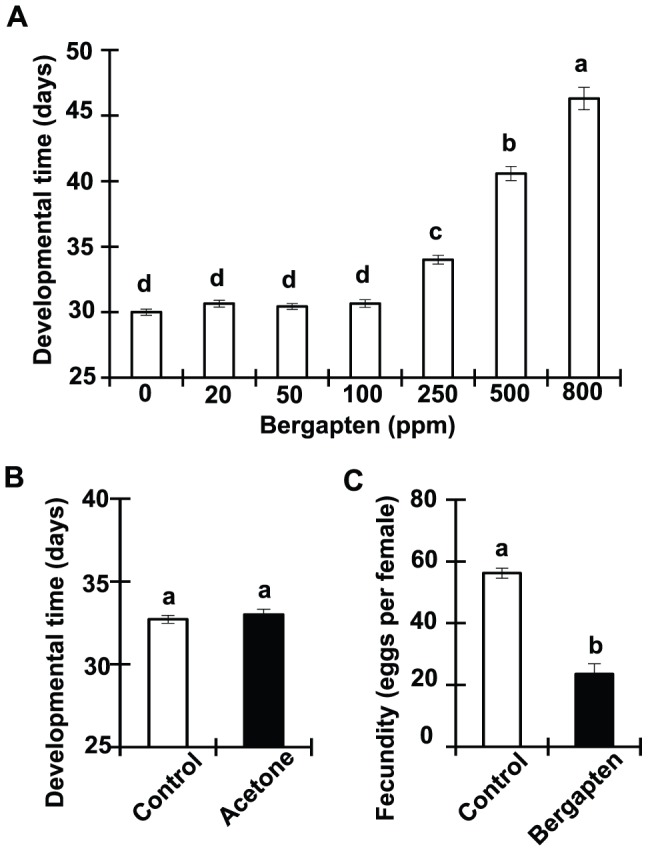
Dietary bergapten negatively affects cowpea bruchid development and fecundity. (A) Developmental time (days, mean±SE) of bruchids when fed bergapten at doses ranging from 0 to 800 ppm as shown. Data were analyzed using a one-way ANOVA (*F*_6, 325_ = 179.6, *P*<0.001). Tukey's multiple range test was used to compare the difference between treatments. Means followed by different letters indicate significant difference between treatments (Tukey test: *P*<0.05). Developmental time is defined as the time period from egg laying to adult emergence. (B) Use of acetone as bergapten solvent has no effect on cowpea bruchid development. Acetone (10%), equivalent of the amount used in making 1,000 ppm bergapten, was mixed with cowpea flour for making artificial seeds, followed by lyophilization. Developmental time data was analyzed by independent *t*-test. Means followed by the same letter are not significantly different (*t*_1, 59_  = 1.682, *P* = 0.098). (C) Fecundity (eggs per female, mean±SE) when fed to diet containing 250 ppm bergapten. Different letters indicate significant difference between treatments (Independent *t* test: *t*_1, 4_  = 8.733, *P* = 0.001).

To determine the effect of furanocoumarins on growth, survival and reproduction of a coleopteran storage insect pest, bergapten was incorporated into artificial seeds and infested them with cowpea bruchids. Initial tests indicated that bergapten at 1,000 ppm and above caused 100% mortality, so its effect on growth was determined at a range from 20 to 800 ppm. As shown in Fig. 1A, bergapten delayed bruchid development in a dose-dependent fashion. It should be noted that acetone, the solvent of bergapten, did not show any effect on insect development at the dose applied (Fig. 1B). Lyophilization presumably led to evaporation of all liquid. Since bergapten at 250 ppm significantly prolonged developmental time but showed no influence on mortality (data not shown), this concentration was selected to evaluate the effect on fecundity. Insects reared on bergapten-containing seeds produced substantially fewer eggs than those reared on control seeds (Fig. 1C). Results clearly indicate that bergapten interfered with the life cycle of the cowpea bruchid.

### Transcriptomic response to dietary bergapten

To gain insight into how dietary bergapten regulates gene expression in the bruchid digestive system, a midgut cDNA microarray platform established previously was used to profile transcriptome response to bergapten. This microarray comprises 20,352 randomly picked cDNA clones from a normalized, non-subtractive cDNA library constructed using cowpea bruchid larvae subjected to various treatments to increase gene representation (for details, see [32]). A total of 915 cDNAs were induced or repressed by two-fold or more (*P*≤0.05) when the bruchid was challenged by dietary bergapten. Sequencing analyses showed that these cDNAs are 847 bp in length on average. Assembly of these sequences generated 543 contigs, of which 388 were singletons (Table S1). Among this bergapten-regulated unigene set, 257 were newly identified sequences (GenBank accession numbers: JK754869-JK755122, JK817577-JK817579). Putative gene function was determined by sequence alignment in BlastX searches at an E-value cutoff of 10^−6^.

**Figure 2:**
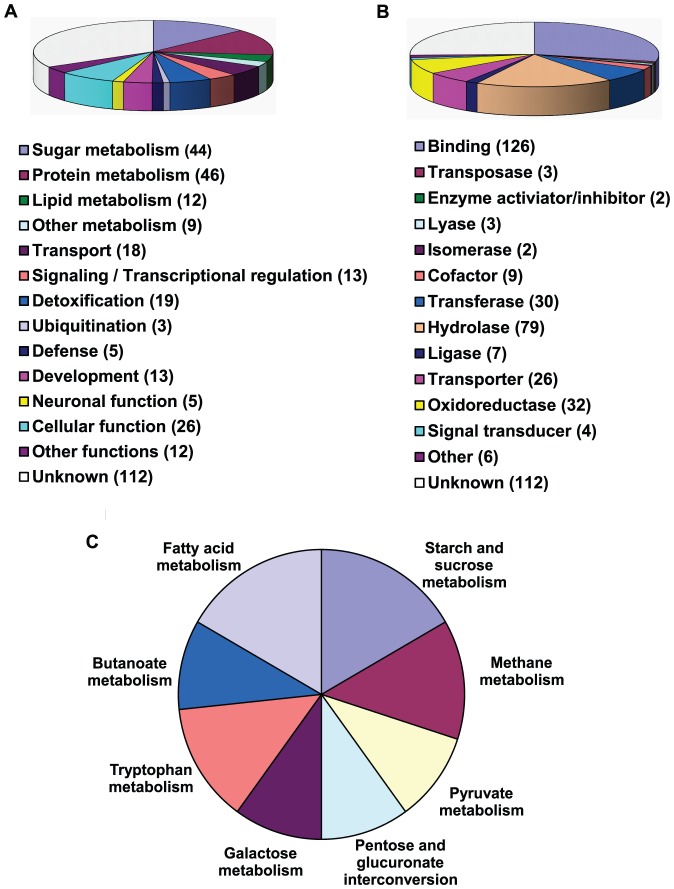
Summary of sequence annotation of bergapten-response genes from cowpea bruchid midgut based on (A) biological function, (B) molecular function and (C) KEGG pathway analyses. The 4^th^ instar larvae reared on artificial diet containing 250 ppm bergapten and control diet, respectively, were removed, their midguts were dissected and total RNA was extracted, followed by microarray hybridization. The BLAST2GO software was used for BlastX search (*E*-value cutoff, 10^−6^) and KEGG pathway mapping of bergapten-responsive genes. Shown are KEGG pathways with at least three genes mapped.

A significant number of contigs (206) had no hits in the Blast search, and 112 matched genes with unknown function. Those (225) that share homology with genes of known function in the database were categorized according to biological process and molecular function (Fig. 2A). Bergapten-regulated genes include those involved in sugar, protein and lipid metabolisms, nutrient transport, development, defense, detoxification, signaling and various cellular functions. Such a broad impact suggests that the insect digestive system actively reallocates genomic resources to mitigate negative effect of dietary bergapten. KEGG metabolic pathway analyses indicated that the most abundant categories for bergapten effect were associated with metabolism of carbohydrates and fatty acids (Fig. 2B).

### Genes antagonistically regulated by bergapten and scN

Comparison of expression profiles of bergapten- and scN-responsive genes revealed some striking features; first, approximately one third of bergapten-regulated midgut unigenes (200) in the 4^th^ instar were also scN-responsive genes [32]. More interestingly, of the 200 scN- and bergapten-coregulated gene set, 195 of them were oppositely regulated by the two chemicals. Eighty of the 200 genes had putative functions (based on Blast matches), and 78 of these 80 were antagonistically regulated by scN and bergapten (Table 1).

**Table 1:** Cowpea bruchid midgut genes coregulated by bergapten and scN.

Category	Accession#	Putative Function (Abbreviation)	Fold change^a^	
			Bergapten	scN
Sugar Metabolism				
	FK668918	alpha-Amylase (AMY1)	0.43	2.14
	FK668899	alpha-Glucosidase (AGL1)	0.26	2.90
	FK668900	alpha-Glucosidase (AGL2)	0.47	2.46
	FK668901	alpha-Glucosidase (AGL3)	0.37	3.05
	FK668902	alpha-Glucosidase (AGL4)	0.18	2.11
	FK668936	beta-1,4-Mannanase 1 (MAN2)	0.13	3.69
	FK668881	beta-Galactosidase (BGL2)	0.46	9.28
	FK668883	beta-Galactosidase (BGL4)	0.40	5.41
	FK668907	beta-Glucosidase (BGA4)	0.40	2.18
	FK668908	beta-Glucosidase (BGA5)	0.27	2.48
	FK668909	beta-Glucosidase (BGA6)	0.11	2.07
	FK668910	beta-Glucosidase (BGA7)	0.49	4.52
	FK668911	beta-Glucosidase (BGA8)	0.43	6.19
	FK668914	beta-Glucosidase (BGA11)	0.41	3.61
	FK668915	beta-Glucosidase (BGA12)	0.25	2.37
	FK669587	Glycoside hydrolase family protein 5 (GH5)	0.16	3.81
	GW917132	Glycoside hydrolase family protein 28 (GH28-1)	0.23	2.28
	FK668916	Glycosyl hydrolase family 31 protein (GH31-1)	0.38	2.51
	FK668917	Glycosyl hydrolase family 31 protein (GH31-2)	0.32	2.64
	GW917355	Mitochondrial enolase superfamily member 1 (ENOSF1b)	0.29	6.95
	FK668996	Pectate lyase (PEL2)	0.45	2.02
	FK668897	beta-Mannosidase A (MANBA)	8.65	0.01
Protein metabolism				
	FK668971	Carboxypeptidase, vitellogenic-like (CPVL1)	0.37	3.79
	FK668961	Cathepsin B (CatB6)	0.19	3.40
	FK668962	Cathepsin B (CatB7)	0.31	2.20
	FK668948	Cathepsin L (CatL1)	0.36	2.39
	FK668951	Cathepsin L (CatL4)	0.27	4.90
	FK668952	Cathepsin L (CatL5)	0.29	4.03
	FK668953	Cathepsin L (CatL6)	0.32	3.77
	FK669001	Cystathionine beta-lyase (CBL2)	6.00	0.45
	FK669322	Eukaryotic translation initiation factor 4 gamma 2 (eIF4G2-1)	2.53	0.44
	FK669004	Glutaminyl-peptide cyclotransferase (QPCT)	0.47	2.84
	FK669330	HBS1/Elongation factor 1 alpha-like protein (HBS1)	6.94	0.09
	FK669008	Homocysteine S-methyltransferase (HMT)	2.28	0.46
	FK669005	Phosphoserine aminotransferase (PSAT1-1)	3.35	0.24
	FK669006	Phosphoserine aminotransferase (PSAT1-2)	2.00	0.34
	FK669007	Phosphoserine aminotransferase (PSAT1-3)	3.13	0.25
	FK668974	Plasma glutamate carboxypeptidase (PGCP1)	0.39	2.70
	FK668976	Plasma glutamate carboxypeptidase (PGCP3)	0.49	3.22
	FK669010	Prolyl-4-hydroxylase-alpha EFB (PH4alphaEFB1)	4.31	0.02
	FK669011	Prolyl-4-hydroxylase-alpha EFB (PH4alphaEFB2)	4.71	0.02
	FK668967	Retinoid-inducible serine carboxypeptidase (RISC2)	0.46	2.04
	FK668979	Trypsinogen RDOT3 (RDOT3)	0.35	3.34
Lipid metabolism				
	FK669017	24-Dehydrocholesterol reductase (DHCR24-1)	0.45	2.31
	FK669019	24-Dehydrocholesterol reductase (DHCR24-3)	0.49	2.29
	FK669021	Aldo-keto reductase (AKR1)	0.35	2.48
	FK669046	Glucosylceramidase (GBA)	0.47	4.14
Other metabolism				
	FK669060	5-Oxoprolinase (ATP-hydrolysing) (OPLAH)	0.44	2.20
	FK669054	Glyoxylate reductase hydroxypyruvate reductase (GRHPR)	0.44	3.52
Transport				
	FK669075	Solute carrier family facilitated glucose transporter member 8 (GLUT1)	0.45	2.63
	FK669446	Solute carrier family facilitated glucose transporter member 8 (GLUT3)	2.31	0.18
	*FK669068*	*Sugar transporter (SUT1)*	2.17	2.27
	FK669069	Sugar transporter (SUT2)	0.45	2.26
	FK669103	Vacuolar H ATPase 100-2 (VHA100-2)	3.27	0.04
Signaling/Transcriptional regulation				
	FK669149	Klotho (Klotho)	0.12	2.62
Detoxification				
	FK669179	Catalase (CAT1)	2.85	0.24
	FK669180	Catalase (CAT2)	3.01	0.31
	FK669165	Cytochrome P450 (CYP6G1-2)	4.65	0.39
	FK669166	Cytochrome p450 (CYP6G1-3)	8.06	0.37
	FK669192	Esterase-6 precursor (EST2)	0.43	2.23
	FK669177	Glutathione S-transferase (GST)	3.48	0.48
	*FK669176*	*Peroxidase precursor (POD)*	0.20	0.32
Ubiquitination				
	FK669204	Ubiquitin-conjugating enzyme E2-17 kDa (E2(17)KB)	2.50	0.35
Defense				
	FK669230	Drosomycin-like I (DrsL1-1)	18.97	0.03
	FK669231	Drosomycin-like I (DrsL1-2)	21.75	0.02
Development				
	FK669269	Extramacrochaetae protein (EMC)	2.11	0.41
	FK669260	Juvenile hormone esterase (JHE7)	0.36	2.04
	FK669270	Laccase 2 (LAC2)	3.40	0.09
Neuronal function				
	FK669050	AMP-dependent CoA ligase (CL)	2.33	0.15
	FK669281	Synaptic vesicle membrane protein VAT-1 homolog (VAT1-1)	2.03	0.21
	FK669282	Synaptic vesicle membrane protein VAT-1 homolog (VAT1-2)	2.18	0.12
Cellular function				
	FK669309	Actin binding protein (ABP2)	3.59	0.15
	FK669368	CD9 antigen (CD9-2)	3.41	0.32
	FK669316	Failed axon connections (FAX)	2.72	0.14
	FK669354	Guanosine monophosphate reductase (GMPR)	2.13	0.17
	FK669332	LIM domain protein (LIM2)	2.32	0.17
	FK669357	Lysosomal acid phosphatase 2 (LAP1)	0.46	9.50
	FK669306	Tubulin-specific chaperone a (TBCA)	2.08	0.42
	FK669348	40 kDa salivary protein SP11 (SP11)	0.16	5.92
Other functions				
	FK669624	BCL2 adenovirus e1b 19 kda protein-interacting (BNIP3L)	2.03	0.36

a : Numbers shaded gray indicate genes down-regulated by two-fold or more (P≤0.05) in response to dietary bergapten or scN, Unshaded are up-regulated, and underlined indicate the genes are induced or repressed by both bergapten or scN.

A number of detoxification genes were up-regulated by bergapten but down-regulated by scN. Increasing production of P450s and GSTs, the phase I and II metabolic enzymes, respectively, has been recognized as one of the important counter defense mechanisms that insects use for plant allelochemicals [10], [35]. Catalases (CAT) play a pivotal role in detoxification and cellular defense against oxidative stress [17], [18]. Laccase 2 (*LAC2*), a phenoloxidase gene [36], [37] was also induced in bergapten-fed bruchids. Presumably oxidative crosslinking may help decrease bergapten penetration of insect midgut. Furthermore, among bergapten-induced genes were several that are associated with disease resistance (Table S1). The drosomycin-like genes are of central importance in the insect immune system against bacterial infection [38]. Notably, they are also repressed by scN (Table 1). On the contrary, many genes involved in protein, lipid and carbohydrate digestion were suppressed under bergapten treatment, opposite to their responses to scN.

### scN potentiates bergapten anti-insect effect

**Figure 3:**
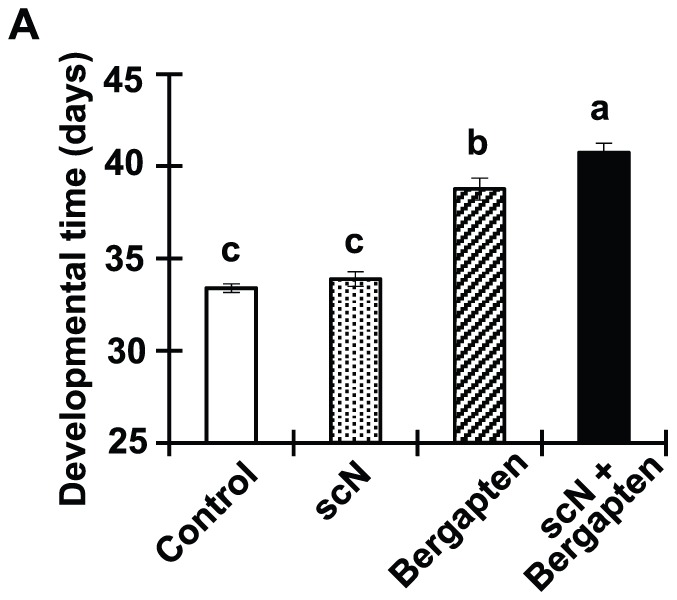
scN potentiates the anti-insect effect of bergapten. Developmental time (days, mean±SE) of bruchids when fed the control diet and diet containing 1,000 ppm scN, 250 ppm bergapten, or 1000 ppm scN + 250 ppm bergapten, respectively, was analyzed by one-way ANOVA (*F*_3, 128_ = 68.1, *P*<0.001). Tukey's multiple range test was used to compare the difference between treatments. Means followed by different letters indicate significant difference between treatments (Tukey test: *P*<0.05). Developmental time is defined as Fig. 1.

The antagonistic effect of scN and bergapten on the vast majority of coregulated genes is intriguing. Possibly, scN is able to prevent bergapten-induced adaptive transcriptional adjustment, and even to synergize bergapten's anti-insect activity. To investigate the potential interaction between bergapten and scN in the insect midgut, a combination of both chemicals were incorporated into the artificial seeds to test their effect on development of the insect. Bergapten at 250 ppm exhibited a mild but significant effect on the development of the bruchids. scN at 1,000 ppm showed no significant effect by itself, yet further delayed bruchid development by 2 days in the combination treatment when compared to the bergapten treatment (Fig. 3).

**Figure 4:**
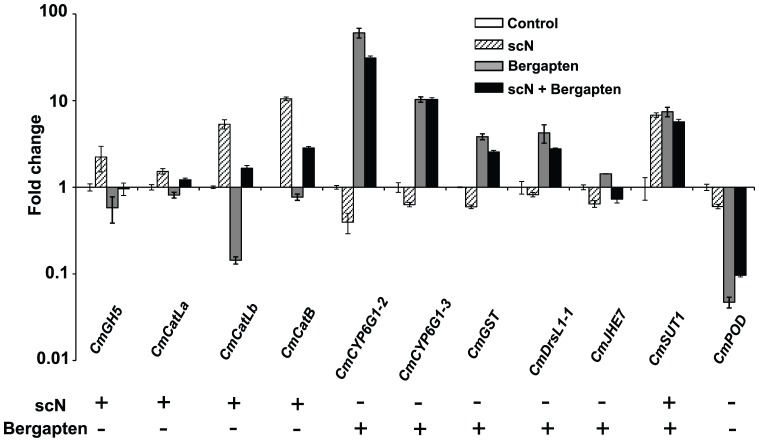
scN attenuates transcriptional changes induced by bergapten. Selected bergapten- and scN-coregulated genes involved in polysaccharide or protein degradation (*CmGH5*, *CmCatL*s, *CmCatB*), detoxification (*CmCYP6G1*s, *CmGST*, *CmPOD*), defense (*CmDrsL1-1*), development (*CmJHE7*) and transport (*CmSUT1*) were subjected to qPT-PCR analyses. Total RNA was extracted from midgut of the 4^th^ instar larvae feeding on artificial diet containing 1,000 ppm scN, 250 ppm bergapten or 1,000 ppm scN + 250 ppm bergapten, respectively. Insects feeding on diet without bergapten or scN served as the control. Reverse transcription and qRT-PCR reactions were performed as described in Material and Methods. Transcript fold induction derived from qRT-PCR is shown as bar graphs. The lower panel shows microarray results of the corresponding genes. “+”, “−”: up- or down-regulation when subjected to scN or bergapten treatment. *CmGH5*, Glycoside hydrolase; *CmCatLa* and *CmCatLb*, cathepsin L-like proteases; *CmCatB*, cathepsin B-like protease; *CmCYP6G1-2* and *CmCYP6G1-3*, Cytochrome P450s; *CmGST*, Glutathione S-transferase; *CmDrsL1-1*, Drosomycin-like I; *CmJHE7*, Juvenile hormone esterase; *CmSUT1*, Sugar transporter 1; *CmPOD*, Peroxidase precursor.

qRT-PCR of 11 selected genes validated the microarray results associated with bergapten treatment (Fig. 4). Although scN alone, at the concentration we chose, did not cause any apparent change in insect development, it was sufficient to alter transcript abundance of the selected genes, the profile of which is in agreement with cDNA microarray results (Fig. 4). These bergapten- and scN- coregulated genes include those potentially involved in carbohydrate and protein degradation, disease resistance, and stress tolerance. Antagonistic regulation has been observed for many of these genes, that is, genes induced by scN but repressed by bergapten, or vice versa, had much lowered induction or suppression in the combinatory treatment compared to individual chemical treatments (Fig. 4). Since insect adaptation to dietary challenges is at least in part mediated by transcriptional regulation, attenuation of bergapten-induced transcriptional adjustment by scN among genes of broad functionality could be responsible for their synergistic anti-insect activity. Out of 80 coregulated genes with Blast hits, the only two that showed the same transcript regulation by scN and bergapten did not result in further induction (in the case of *CmSUT1*) or suppression (in the case of *CmPOD*).

## Discussion

Deploying secondary metabolites is a common defense mechanism used by many plant species to fight against insect herbivory. Here, we tested dietary bergapten for its effect on a coleopteran storage pest and found that it negatively impacted development, reproduction and survival of cowpea bruchids (Fig. 1). Despite the notable plant protective role, many herbivorous insects have evolved various resistance strategies to evade plant defense, and the front line of battle is the digestive canal. Consistently, our microarray analyses demonstrated that the midgut gene expression program in cowpea bruchids changes in response to dietary challenges, whether the challenge was derived from scN [32] or bergapten (Fig. 2). Genome-wide resource reallocation is perhaps an accommodation essential for insect survival. Among bergapten-induced genes are *P450*, *GST*, *CAT* and *LAC2* genes (Table S1), known to encode allelochemical-detoxifying enzymes that confer metabolic resistance, or phenoloxidase that possibly increases resistance to penetration of bergapten [10], [18], [37]. Thus it is a logical assumption that transcriptomic reconfiguration contributes to mitigating the toxic effect of plant defensive metabolites.

While some P450 genes are induced, others are down-regulated (Table S1). This differential response possibly reflects the large number, different substrate specificity and diverse functions that P450s may possess in cowpea bruchids. Bergapten can inhibit the housefly *CYP6D1* gene, while it also serves as the substrate of other P450s [39]. In addition to detoxification of xenobiotics including plant secondary metabolites and synthetic pesticides, P450s control synthesis and degradation of insect hormones, and thus play important roles in insect growth and development [40], [41]. Delayed development in response to dietary bergapten is in agreement with altered development regulatory genes, such as JH esterases or JH epoxide hydrolases (Table S1).

It is well established that furanocoumarins induce phototoxicity in many insects that feed on diets containing these compounds, by producing reactive oxygen species [5], [8], [42]. Although cowpea bruchid larvae feed inside the seeds and are therefore shielded from most direct light, shelf light in our insect-rearing chamber could have penetrated the seeds to a certain extent and resulted in some levels of oxidative stress. The notion that phototoxicity may have contributed to the overall anti-insect activity we observed is supported by the induction of *LAC2* by bergapten (Table 1). LAC2 is required for cuticle sclerotization and egg chorion tanning. Protection from phototoxins by pigmentation has been known to occur in mammals and in insects [1]. Induction of cowpea bruchid *LAC2* could result from insect response to phototoxicity, although furanocoumarins are antifeedants to insects even in the absence of light [1], [42].

One of the highly induced genes revealed by our microarray study encodes the antifungal peptide drosomycin [43], suggesting the involvement of insect immunity in response to dietary challenges. Cross-talk between immune response and other stress responses, and pathway convergence in general, is becoming an emerging theme that has been discussed recently in both vertebrates and invertebrates [44]–[49]. For instance, starvation can induce antimicrobial peptide genes in non-infected or immunity-defective insects through activating a transcription factor FOXO, independent of pathogen responsive pathways [46]. Similarly, salt stress or high oxygen environment induces immune gene expression [47], [50]. In our study, bergapten-induced increase in the reactive oxygen species level could be responsible for activation of drosomycin genes. It has been shown that antimicrobial peptide production, whether induced through immunity-dependent or -independent pathway, enhances tolerance of animals to oxidative stress. Induction of drosomycin thus likely facilitates cowpea bruchids' coping with dietary bergapten.

Although altered gene expression in response to dietary challenge allows insects to more effectively utilize the available resources, this often compromises other unchallenged physiological systems. As shown in cowpea bruchids, enhanced protein and carbohydrate digestion induced by scN is accompanied by reduced detoxification and stress tolerance. Likewise, higher expression of detoxification genes to increase metabolic resistance as a result of bergapten treatment comes at a cost of lower food digestibility, reflected by the decreased expression of protease and α-amylase expression. Such a trade-off could be viewed as a vulnerability that can be exploited for designing synergistic anti-insect agents.

We have previously demonstrated the combinatorial effect of scN and wheat α-amylase inhibitor on cowpea bruchids [51]. The α-amylase inhibitor has very limited impact when ingested alone due to its protease-sensitive nature. But this impact was greatly enhanced when fed to insects together with scN. Inhibition of protease activity by scN not only limited the availability of free amino acids necessary for insect growth, but prevented α-amylase inhibitor from proteolysis by gut digestive enzymes, resulting in increased effective concentration of α-amylase inhibitor. Such synergistic insecticidal activity has also been observed when scN is administered together with aspartic protease inhibitor pepstatin, or with Kunitz trypsin inhibitor [52]. Protecting protease-sensitive but otherwise toxic proteins in the insect digestive canal and ensuring that they reach their target sites apparently is effective in maximizing plant defense. Not all chemical combinations, however, lead to synergism. Additive or even antagonistic effects on insect mortality have been observed when bergapten was mixed with xanthotoxin and psoralen, respectively [5].

The most intriguing finding in our current study resulted from the profiling of bergapten- and scN-coregulated genes (Table 1). Feeding cowpea bruchids with scN + bergapten resulted in much more delayed insect development compared to bergapten alone (Fig. 3). Transcriptomic profiling revealed that a significant number of midgut genes respond oppositely to scN and bergapten challenges (Table 1). When combined, bergapten and scN interfere with each other's transcriptional responses (Fig. 4), potentially explaining their synergism. We have previously shown that cowpea bruchids could adapt to dietary scN by over-producing major digestive cathepsin L-like proteases as well as by activating scN-insensitive cathepsin B-like proteases [31], [32]. Transcriptional activation of these counter-defense genes was substantially attenuated in the combination treatment (Fig. 4). Similarly, induction of some detoxification genes by bergapten alone was compromised when scN was also present in the diet. It is interesting that most coregulated genes are still induced in the combination treatment. Possibly the bruchids attempted to adjust to the presence of both chemicals, but obviously at a lower capacity. Therefore, adaptation strategies that are sufficient when insects are dealing with individual defense compounds may not be effective when insects are challenged by both compounds simultaneously.

From a pest control perspective, as single plant defense compounds or single resistance gene products often fail to give adequate protection against insect pests due to rapid development of insect adaptation, combining plant defensive compounds that have opposite impacts on expression of adaptation-related genes may represent a strategy to increase anti-insect activity. Antagonistic gene regulation by the treatment compounds, as shown in the scN + bergapten treatment, decreased fold changes of adaptation-related genes. As a result, insects' ability to overcome either active molecule is prevented or at least weakened, potentially increasing effectiveness of the defense compounds.

Resistance development in agriculture is an ongoing challenge to pest management. Genes associated with resistance of insects to plant secondary compounds are thought to share an evolutionary association with genes involved in synthetic insecticide detoxification [10], [53]. The results from this study should provide some insight into effective pesticide usage. Discovering synergists of effective insecticides could potentially further increase anti-insect efficacy, and at the same time delay resistance development.

## Supporting Information

Table S1 Bergapten-responsive genes identified by microarray from cowpea bruchid alimentary tract.
(DOC)
