# Supplementary material for: Antagonistic Regulation, Yet Synergistic Defense: Effect of Bergapten and Protease Inhibitor on Development of Cowpea Bruchid Callosobruchus maculatus
Source: PLoS One. 2012 Aug 21;7(8):e41877. doi: 10.1371/journal.pone.0041877 (PMC3424127; doi:10.1371/journal.pone.0041877)
Supplement: Table S1 — Bergapten-responsive genes identified by microarray from cowpea bruchid alimentary tract. (DOC) [file pone.0041877.s001.doc]

**Supplementary Table S1.** Bergapten-responsive genes identified by microarray from cowpea bruchid alimentary tract.

| **Category** | **Accession#** | **Tribolium homolog** | **Abbreviation** | **Fold change^a^** | **Putative Gene Identification^b^** |
| --- | --- | --- | --- | --- | --- |
| Sugar Metabolism | | | | | |
|  | FK668941 | XP_970690.2 | AEP1 | 0.32 | Aldose 1-epimerase |
|  | FK668899 | XP_967022.2 | AGL1 | 0.26 | Alpha-glucosidase |
|  | FK668900 | XP_975228.1 | AGL2 | 0.47 |  |
|  | FK668901 | XP_967022.2 | AGL3 | 0.37 |  |
|  | FK668902 | XP_967022.2 | AGL4 | 0.18 |  |
|  | FK668918 | XP_969234.1 | AMY1 | 0.43 | Alpha amylase |
|  | JK755020 | XP_969380.1 | AMY7 | 0.35 |  |
|  | JK755026 | XP_969234.1 | AMY8 | 0.36 |  |
|  | FK668904 | XP_972032.1 | BGA1 | 0.32 | Beta-glucosidase |
|  | FK668906 | XP_972437.1 | BGA3 | 0.15 |  |
|  | FK668907 | XP_972437.1 | BGA4 | 0.40 |  |
|  | FK668908 | XP_972437.1 | BGA5 | 0.27 |  |
|  | FK668909 | XP_975666.1 | BGA6 | 0.11 |  |
|  | FK668910 | XP_975653.1 | BGA7 | 0.49 |  |
|  | FK668911 | XP_972082.1 | BGA8 | 0.43 |  |
|  | FK668914 | XP_972437.1 | BGA11 | 0.41 |  |
|  | FK668915 | XP_972032.1 | BGA12 | 0.25 |  |
|  | JK755087 | XP_972437.1 | BGA14 | 0.22 |  |
|  | JK755117 | XP_972082.1 | BGA15 | 0.09 |  |
|  | GW917103 | XP_972437.1 | BGA16 | 0.39 |  |
|  | FK668881 | XP_967647.1 | BGL2 | 0.46 | Beta-galactosidase |
|  | FK668883 | XP_967647.1 | BGL4 | 0.40 |  |
|  | GW917355 | XP_972041.1 | ENOSF1b | 0.29 | Mitochondrial enolase superfamily member 1 |
|  | JK755044 | XP_971568.1 | FUCA1 | 0.37 | Plasma alpha-l-fucosidase |
|  | JK755091 | XP_974181.1 | GAPDH | 0.47 | Glyceraldehyde-3-phosphate dehydrogenase |
|  | GW917132 |  | GH28-1 | 0.23 | Glycoside hydrolase family protein 28 |
|  | JK755039 |  | GH28-2 | 0.22 |  |
|  | FK668916 | XP_972326.1 | GH31-1 | 0.38 | Glycosyl hydrolase family 31 protein |
|  | FK668917 | XP_972326.1 | GH31-2 | 0.32 |  |
|  | FK669587 |  | GH5 | 0.16 | Glycoside hydrolase family protein 5 |
|  | GW917119 | XP_969423.1 | GUSB | 0.46 | Beta-glucuronidase |
|  | FK668926 | XP_970182.1 | L-GALDH | 2.20 | L-galactose dehydrogenase |
|  | FK668936 |  | MAN2 | 0.13 | Beta-1,4-mannanase 1 |
|  | FK668897 | XP_974383.1 | MANBA | 8.65 | Beta-mannosidase A |
|  | JK755032 | XP_975546.1 | MDH1 | 0.32 | Malate dehydrogenase |
|  | JK755040 | XP_973533.1 | MDH2 | 0.49 |  |
|  | JK755097 | XP_975546.1 | MDH3 | 0.49 |  |
|  | JK755034 | XP_973533.1 | MDH4 | 0.46 |  |
|  | FK668943 | XP_001813448.1 | NPL | 0.38 | N-acetylneuraminate pyruvate lyase |
|  | FK668996 |  | PEL2 | 0.45 | Pectate lyase |
|  | GW917132 |  | PEL7 | 0.32 |  |
|  | FK668940 | XP_968140.1 | PGK1 | 0.38 | Phosphoglycerate kinase |
|  | FK669455 | XP_966733.2 | PPS | 0.47 | Phosphoenolpyruvate synthase |
|  | JK755108 | XP_966585.1 | TALDO | 0.48 | Transaldolase |
| Protein metabolism | | | | | |
|  | FK669013 | XP_966408.1 | AGXT2L1 | 0.28 | Alanine-glyoxylate aminotransferase 2-like 1 |
|  | GW917153 | XP_969482.1 | ALG | 2.06 | Alpha-1,2-mannosyltransferase |
|  | GW917285 | XP_001811586.1 | CALR | 0.32 | Calreticulin |
|  | FK668961 | NP_001164205.1 | CatB6 | 0.19 | Cathepsin B |
|  | FK668962 | XP_974298.1 | CatB7 | 0.31 |  |
|  | FK668948 | NP_001164314.1 | CatL1 | 0.36 | Cathepsin L |
|  | FK668951 | NP_001164314.1 | CatL4 | 0.31 |  |
|  | FK668952 | NP_001164088.1 | CatL5 | 0.29 |  |
|  | FK668953 | NP_001164088.1 | CatL6 | 0.32 |  |
|  | JK755027 | XP_970644.1 | CatL14 | 0.31 |  |
|  | JK755118 | XP_970644.1 | CatL15 | 0.37 |  |
|  | JK755064 | XP_970773.1 | CatL16 | 0.48 |  |
|  | JK755074 | NP_001164314.1 | CatL17 | 0.31 |  |
|  | JK755085 |  | CatL18 | 0.30 |  |
|  | JK755104 | NP_001164314.1 | CatL19 | 0.31 |  |
|  | JK755111 | XP_970644.1 | CatL20 | 0.31 |  |
|  | FK669001 | XP_972447.1 | CBL2 | 6.00 | Cystathionine beta-lyase |
|  | FK668971 | XP_969249.1 | CPVL1 | 0.37 | Carboxypeptidase, vitellogenic-like |
|  | JK755076 | XP_969249.1 | CPVL2 | 0.36 |  |
|  | JK755084 | XP_969249.1 | CPVL3 | 0.36 |  |
|  | JK755043 | NP_001107835.1 | EF1alpha | 0.49 | Elongation factor 1 alpha |
|  | JK755036 | XP_001809758.1 | EF1gamma | 0.43 | Elongation factor 1-gamma |
|  | FK669322 | XP_969772.2 | eIF4G2-1 | 2.53 | Eukaryotic translation initiation factor 4 gamma 2 |
|  | FK669330 | XP_968959.1 | HBS1 | 6.94 | HBS1/Elongation factor 1 alpha-like protein |
|  | FK669008 | XP_966501.1 | HMT | 2.28 | Homocysteine S-methyltransferase |
|  | JK755078 |  | NPEP | 0.45 | Aminopeptidase N |
|  | JK755072 | XP_969003.1 | OST1 | 0.39 | Oligosaccharyl transferase |
|  | JK755114 | XP_971377.1 | OST2 | 0.50 |  |
|  | JK755096 | XP_972341.1 | OST3 | 0.49 |  |
|  | JK755113 | XP_971685.1 | PDI | 0.39 | Protein disulfide-isomerase |
|  | FK668974 | XP_971371.1 | PGCP1 | 0.39 | Plasma glutamate carboxypeptidase |
|  | FK668976 | XP_971371.1 | PGCP3 | 0.49 |  |
|  | FK669010 | XP_969386.1 | PH4alphaEFB1 | 4.31 | Prolyl-4-hydroxylase-alpha EFB |
|  | FK669011 | XP_969386.1 | PH4alphaEFB2 | 4.71 |  |
|  | FK669005 | XP_974471.1 | PSAT1-1 | 3.35 | Phosphoserine aminotransferase |
|  | FK669006 | XP_974471.1 | PSAT1-2 | 2.00 |  |
|  | FK669007 | XP_974471.1 | PSAT1-3 | 3.13 |  |
|  | FK669004 | XP_970809.1 | QPCT | 0.47 | Glutaminyl-peptide cyclotransferase |
|  | FK668979 | XP_001814174.1 | RDOT3 | 0.35 | Trypsinogen RDOT3 |
|  | FK668966 | XP_969249.1 | RISC1 | 0.48 | Retinoid-inducible serine carboxypeptidase |
|  | FK668967 | XP_969249.1 | RISC2 | 0.46 |  |
|  | JK755101 | XP_969249.1 | RISC4 | 0.29 |  |
|  | JK755069 |  | TLP3 | 0.47 | Trypsin-like serine proteinase |
|  | JK755067 | XP_969332.2 | UGGT | 0.44 | UDP-glucose:glycoprotein glucosyltransferase |
|  | JK755038 | XP_971015.1 | γGH1 | 0.50 | Gamma-glutamyl hydrolase |
|  | JK755042 | XP_971015.1 | γGH2 | 0.47 |  |
| Lipid metabolism | | | | | |
|  | FK669021 | XP_969456.1 | AKR1 | 0.35 | Aldo-keto reductase |
|  | JK755019 | XP_969456.1 | AKR4 | 0.37 |  |
|  | JK755041 | XP_969526.1 | AKR5 | 0.44 |  |
|  | FK669050 | XP_966640.1 | CL | 2.33 | AMP dependent CoA ligase |
|  | FK669017 | XP_001810773.1 | DHCR24-1 | 0.45 | 24-dehydrocholesterol reductase |
|  | FK669019 | XP_001810773.1 | DHCR24-3 | 0.49 |  |
|  | FK669046 | XP_975649.1 | GBA | 0.47 | Glucosylceramidase |
|  | GW917144 | XP_975143.1 | HACL | 0.41 | 2-hydroxyphytanoyl-CoA lyase |
|  | JK755086 | XP_973530.1 | HADH | 0.46 | 3-hydroxyacyl-CoA dehyrogenase |
|  | JK755035 | XP_971720.1 | HSD17B12 | 0.46 | Estradiol 17-beta-dehydrogenase 12 |
|  | FK669048 | XP_973103.2 | LIP3-1 | 0.29 | Lipase 3 |
|  | JK817577 | XP_973103.2 | LIP3-2 | 0.33 |  |
| Other metabolism | | | | | |
|  | JK755057 | XP_968905.1 | ADH1 | 2.75 | Alcohol dehydrogenase |
|  | JK755023 | XP_968905.1 | ADH2 | 2.35 |  |
|  | JK755050 | XP_968925.2 | ALP | 2.16 | Alkaline tissue-nonspecific isozyme-like (alkaline phosphatase) |
|  | GW917159 | XP_972630.1 | COQ5 | 0.39 | Ubiquinone biosynthesis methyltransferase |
|  | FK669054 | XP_973278.1 | GRHPR | 0.44 | Glyoxylate reductase hydroxypyruvate reductase |
|  | JK755060 | XP_972461.1 | NAPRT1 | 2.10 | Nicotinate phosphoribosyltransferase-like isoform 1 |
|  | FK669060 | XP_970328.1 | OPLAH | 0.44 | 5-oxoprolinase (ATP-hydrolysing) |
|  | JK755112 | XP_971620.2 | SUOX | 0.41 | Sulfite oxidase, mitochondrial-like isoform 1 |
|  | JK755115 | XP_967970.1 | VNN1 | 0.49 | Vanin-like protein 1 |
| Transport | | | | | |
|  | JK755073 | XP_966371.1 | AAT | 0.38 | Amino acid transporter |
|  | FK669075 | XP_966831.1 | GLUT1 | 0.45 | Solute carrier family facilitated glucose transporter member 8 |
|  | FK669446 | XP_974394.1 | GLUT3 | 2.31 |  |
|  | FK669114 | XP_966588.1 | NADHD | 0.48 | NADH dehydrogenase |
|  | FK669098 | XP_968752.1 | NCX | 0.43 | Na-Ca exchanger 5 |
|  | JK755052 | XP_971069.1 | SAT | 2.60 | Sulfate transporter |
|  | JK755022 | XP_970689.1 | SBP | 2.84 | Selenium-binding protein |
|  | FK669080 | XP_967023.2 | SLIF | 0.39 | Cationic amino acid transporter slimfast |
|  | GW917210 | XP_967096.1 | SNX30 | 0.30 | Sorting nexin family member 30 |
|  | FK669068 | XP_973264.1 | SUT1 | 2.17 | Sugar transporter |
|  | FK669069 | XP_974346.1 | SUT2 | 0.45 |  |
|  | JK755068 | XP_969244.1 | TWIK2 | 0.37 | T family of potassium channels family |
|  | JK755018 | XP_976188.1 | VATPase3 | 0.37 | ATP synthase alpha subunit vacuolar |
|  | JK755106 | XP_976188.1 | VATPase4 | 0.50 |  |
|  | JK755033 | NP_001161226.1 | VATPase5 | 0.47 |  |
|  | JK755105 | XP_967959.1 | VATPase6 | 0.50 |  |
|  | FK669103 |  | VHA100-2 | 3.27 | Vacuolar H ATPase 100-2 |
|  | JK755059 | XP_973445.1 | VIP36 | 2.03 | Vesicular integral-membrane protein vip36 |
| Signaling / Transcriptional regulation | | | | | |
|  | JK755102 | XP_971800.2 | AK | 0.48 | Arginine kinase |
|  | JK755061 | XP_975145.2 | CDC2 | 2.02 | Cyclin-dependent kinase |
|  | JK755094 | XP_975731.1 | Galpha | 0.50 | G protein alpha subunit |
|  | FK669149 | XP_968318.1 | Klotho | 0.12 | Klotho |
|  | JK755048 | XP_966347.1 | MBF1 | 2.01 | Multiprotein bridging factor 1 |
|  | GW917232 | XP_973904.1 | NCL-1 | 17.12 | B-box type zinc-finger protein ncl-1 |
|  | JK755099 | EFA01970.1 | PKA | 0.42 | Protein kinase A |
|  | JK755110 | XP_974043.1 | RAB1 | 0.45 | Rab-related protein 1 |
|  | JK755081 | XP_968508.1 | RAB5 | 0.48 | Rab-related protein 5 |
|  | JK755077 | XP_973579.1 | RACK | 0.48 | Receptor for activated protein kinase |
|  | JK755051 | XP_966880.1 | SPC18 | 2.15 | Signal peptidase 18 kda subunit |
|  | JK755024 | XP_974921.1 | TFP1 | 2.24 | Nuclear transcription factor Tfp1 |
|  | JK755047 | EFA00396.1 | ZNF3 | 2.13 | zz-type zinc finger-containing protein 3-like |
| Detoxification | | | | | |
|  | FK669179 | NP_001153712.1 | CAT1 | 2.85 | Catalase |
|  | FK669180 | NP_001153712.1 | CAT2 | 3.01 |  |
|  | FK669196 | XP_001814500.1 | CE1 | 0.44 | Carboxylesterase |
|  | JK755063 | XP_970561.1 | CYP345A1 | 3.05 | Cytochrome P450 |
|  | JK755120 | XP_970561.1 | CYP345A1-2 | 2.43 |  |
|  | JK755122 | EFA12532.1 | CYP345D2 | 2.15 |  |
|  | JK755093 | XP_969633.1 | CYP6BK13 | 0.45 |  |
|  | JK755079 | XP_975569.1 | CYP6BQ6 | 0.49 |  |
|  | FK669165 | XP_970561.1 | CYP6G1-2 | 4.65 |  |
|  | FK669166 | XP_970561.1 | CYP6G1-3 | 8.06 |  |
|  | JK755053 | XP_970633.1 | CYP6K1 | 2.39 |  |
|  | JK755098 | XP_973619.2 | CYP9AD1 | 0.47 |  |
|  | FK669176 | XP_967241.1 | POD | 0.09 | Peroxidase precursor |
|  | FK669192 | XP_970896.1 | EST2 | 0.43 | Esterase-6 precursor (EST-6) |
|  | FK669193 | XP_972693.2 | EST3 | 0.31 | Esterase |
|  | JK755016 | XP_966349.1 | GCLC | 2.30 | Glutamate-cysteine ligase catalytic subunit-like |
|  | FK669177 | XP_966526.1 | GST | 3.48 | Glutathione S-transferase |
|  | JK755037 | XP_967845.2 | UGT5 | 0.35 | UDP-glucuronosyltransferase |
|  | JK755109 | XP_967762.1 | UGT6 | 0.43 |  |
| Ubiquitination | | | | | |
|  | JK755045 | XP_974579.1 | CUL2 | 2.06 | Cullin- isoform b |
|  | FK669204 | XP_971130.1 | E2(17)KB | 2.50 | Ubiquitin-conjugating enzyme E2-17 kDa |
|  | JK755092 | NP_001164208.1 | SLIMB | 0.44 | F-box/WD40-repeat protein slimb |
| Defense | | | | | |
|  | JK755054 |  | AFP1 | 2.01 | Salivary gland antifungal peptide 1 |
|  | FK669230 |  | DrsL1-1 | 18.97 | Drosomycin-like I (Drs-lI) |
|  | FK669231 |  | DrsL1-2 | 21.75 |  |
|  | JK755065 | XP_968009.1 | IFN | 0.46 | Ribonuclease l inhibitor homolog (interferon) |
|  | JK755119 | NP_001161922.1 | PMP3 | 0.40 | Peritrophic matrix protein 3 precursor |
| Development | | | | | |
|  | FK669273 | XP_974607.1 | CRALBP1 | 0.35 | Cellular retinaldehyde-binding protein |
|  | FK669269 | XP_970015.2 | EMC | 2.11 | Extramacrochaetae protein |
|  | JK755070 | XP_974395.1 | FAMeT | 0.35 | Farnesoic acid o-methyltransferase-like |
|  | JK755095 | XP_966983.1 | GFAT | 0.43 | Glucosamine--fructose-6-phosphate aminotransferase |
|  | FK669260 | XP_970896.1 | JHE7 | 0.36 | Juvenile hormone esterase |
|  | JK755017 | XP_968291.1 | JHE8 | 0.39 |  |
|  | JK755030 | NP_001164097.1 | JHE9 | 0.39 |  |
|  | JK755075 | NP_001164097.1 | JHE10 | 0.45 |  |
|  | FK669267 | NP_001161927.1 | JHEH1-1 | 0.43 | Juvenile hormone epoxide hydrolase 1 |
|  | JK755031 | XP_970843.1 | JHEH1-2 | 0.36 |  |
|  | FK669270 | NP_001034487.2 | LAC2 | 3.40 | Laccase 2 |
|  | JK755080 | XP_968215.1 | PDE | 0.40 | Pheromone-degrading enzyme |
|  | JK755089 | XP_967067.1 | SLE | 0.48 | Slender lobes |
| Neuronal function | | | | | |
|  | FK669286 | XP_966343.1 | GABA-AT1 | 0.46 | 4-aminobutyrate aminotransferase |
|  | FK669287 | XP_966343.1 | GABA-AT2 | 0.42 |  |
|  | JK755088 | XP_970008.1 | SNMP2 | 0.48 | Sensory neuron membrane protein 2 |
|  | FK669281 | XP_971755.1 | VAT1-1 | 2.03 | Synaptic vesicle membrane protein VAT-1 homolog |
|  | FK669282 | XP_971755.1 | VAT1-2 | 2.18 |  |
| Cellular function | | | | | |
|  | FK669357 | XP_971087.2 | LAP1 | 0.46 | Lysosomal acid phosphate 2 |
|  | FK669309 | XP_968496.1 | ABP2 | 3.59 | Actin binding protein |
|  | JK755062 | XP_966729.2 | ALS | 2.41 | 5-aminolevulinic acid synthase |
|  | JK755090 | XP_967645.1 | ATP5G2 | 0.41 | ATP synthase lipid-binding protein, mitochondrial |
|  | JK755121 | XP_001814720.1 | ATP5s | 0.39 | ATP synthase subunit s, mitochondrial |
|  | JK755028 | NP_001164361.1 | ATPase | 0.40 | ATP synthase beta |
|  | JK755083 | NP_001164361.1 | ATPsyn-beta | 0.40 | ATP synthase subunit mitochondrial |
|  | FK669181 | XP_001814047.1 | BH1 | 0.43 | Bleomycin hydrolase |
|  | FK669368 | XP_967238.1 | CD9-2 | 3.41 | CD9 antigen |
|  | FK669290 |  | CDHL1 | 0.18 | Cadherin-like gene |
|  | JK755103 | XP_001812556.1 | CHD | 0.40 | Chromodomain helicase-DNA-binding protein |
|  | FK669316 | XP_968396.2 | FAX | 2.72 | Failed axon connections |
|  | FK669354 | EFA06520.1 | GMPR | 2.13 | Guanosine monophosphate reductase |
|  | FK669343 | XP_975468.1 | H2AFZ1 | 0.47 | H2A histone family, member Z |
|  | JK755058 | EFA07084.1 | HAL | 2.27 | Histidine ammonia-lyase |
|  | FK669332 | XP_970395.2 | LIM2 | 2.32 | LIM domain protein |
|  | GW917112 | XP_973797.1 | ND | 0.33 | NADH-ubiquinone oxidoreductase |
|  | JK755071 | XP_973797.1 | NUOG | 0.45 | NADH-ubiquinone reductase 75 kda subunit |
|  | GW917336 | NP_001164136.1 | PFN | 0.24 | Profilin |
|  | FK669456 | XP_969432.2 | POL | 0.47 | Retrovirus-related pol polyprotein from transposon TNT 1-9 |
|  | JK755056 | EFA13456.1 | POLB2 | 2.03 | DNA pol b2 domain-containing protein |
|  | JK755029 | XP_971634.1 | RPL4 | 0.50 | Ribosomal protein l4 |
|  | GW917345 | XP_974103.1 | SMAP1 | 2.08 | Stromal membrane-associated protein 1 |
|  | FK669348 | XP_970494.1 | SP11 | 0.16 | 40 kDa salivary protein SP11 |
|  | FK669306 | XP_969241.1 | TBCA | 2.08 | Tubulin-specific chaperone a |
|  | JK755107 | XP_974999.1 | WDR48 | 0.41 | WD repeat domain 48 |
| Other functions | | | | | |
|  | FK669624 | XP_973837.1 | BNIP3L | 2.03 | BCL2 adenovirus e1b 19 kda protein-interacting |
|  | FK669215 | XP_967227.1 | CYT c | 0.50 | Cytochrome c |
|  | JK755025 | XP_973874.2 | LUC1 | 2.42 | Luciferin 4-monooxygenase |
|  | JK755116 | XP_969764.1 | LUC2 | 0.45 |  |
|  | JK755055 | XP_973258.1 | MOSC | 2.13 | MOSC domain-containing protein mitochondrial |
|  | JK755049 |  | Transposase, IS1 | 2.22 | Transposase, IS1 family |
|  | JK755046 |  | Transposase, IS4-1 | 2.15 | Transposase, IS4 family |
|  | JK755066 |  | Transposase, IS4-2 | 0.49 |  |
|  | JK755100 |  | Transposase, IS4-3 | 0.47 |  |
|  | JK755021 |  | Transposase2 | 2.28 | Transposase |
|  | JK755082 |  | Transposase3 | 0.48 |  |
|  | GW917446 |  | Transposase4 | 0.41 |  |
| Unknown | | | | | |
|  | FK669235 | XP_972660.1 | 0103$E2 | 2.02 | Unknown |
|  | JK754964 | XP_966349.1 | 0201F12 | 2.28 | Unknown |
|  | JK754986 | XP_968561.1 | 0203C11 | 0.49 | Unknown |
|  | JK754983 | EFA13656.1 | 0204F5 | 0.15 | Unknown |
|  | JK754987 | XP_001814500.1 | 0204H1 | 0.50 | Unknown |
|  | JK754967 | XP_967022.2 | 0204H3 | 0.18 | Unknown |
|  | JK754975 | XP_970253.1 | 0302D8 | 0.20 | Unknown |
|  | FK669023 | XP_970182.1 | 0401F10 | 2.09 | Unknown |
|  | JK755002 | XP_973362.2 | 0403$E5 | 2.23 | Unknown |
|  | JK754981 | EFA11250.1 | 0404A4 | 2.27 | Unknown |
|  | JK754966 | XP_975467.2 | 0501A10 | 2.21 | Unknown |
|  | JK754988 | EFA13462.1 | 0502A12 | 2.12 | Unknown |
|  | JK754982 | XP_969764.1 | 0504B9 | 2.94 | Unknown |
|  | JK755007 | EEZ97394.1 | 0601$E9 | 0.48 | Unknown |
|  | JK754976 | XP_971622.1 | 0901F11 | 0.42 | Unknown |
|  | FK669261 | NP_001120783.1 | 0904B11 | 2.25 | Unknown |
|  | FK669435 | XP_968792.2 | 0904H4 | 2.30 | Unknown |
|  | JK755003 | XP_971001.1 | 1003G3 | 0.48 | Unknown |
|  | JK754961 | XP_973647.1 | 1202H8 | 2.92 | Unknown |
|  | FK669088 | XP_975895.1 | 1301C5 | 3.07 | Unknown |
|  | FK669116 | EEZ98760.1 | 1302F1 | 2.12 | Unknown |
|  | GW917422 | XP_970249.1 | 1302G9 | 2.26 | Unknown |
|  | FK669672 | CAZ39590.1 | 1303D5 | 0.40 | Unknown |
|  | JK755015 | EEZ99728.1 | 1304A11 | 2.06 | Unknown |
|  | FK668987 | EEZ97810.1 | 1401B2 | 0.42 | Unknown |
|  | FK669727 | EFA00758.1 | 1402A9 | 2.26 | Unknown |
|  | FK669331 | XP_970395.2 | 1502A9 | 2.33 | Unknown |
|  | JK755006 | XP_970541.1 | 1601F6 | 0.46 | Unknown |
|  | JK754960 | XP_968396.2 | 1603$E8 | 2.52 | Unknown |
|  | JK754965 | XP_968905.1 | 1802A12 | 2.45 | Unknown |
|  | JK754999 | AAR86938.1 | 1804F11 | 0.32 | Unknown |
|  | FK669147 | XP_002612611.1 | 1901H9 | 34.38 | Unknown |
|  | FK669642 | EFA06203.1 | 1902C11 | 0.49 | Unknown |
|  | FK669298 | XP_970038.1 | 2001D5 | 0.47 | Unknown |
|  | JK754973 | XP_969456.1 | 2001F7 | 0.32 | Unknown |
|  | FK669369 | EFA12829.1 | 2001H7 | 2.96 | Unknown |
|  | JK755013 | XP_974245.1 | 2003H7 | 0.47 | Unknown |
|  | JK754980 | XP_974646.1 | 2004$E1 | 0.46 | Unknown |
|  | FK669361 | XP_967434.1 | 2004G3 | 2.53 | Unknown |
|  | JK755012 | XP_975458.2 | 2104H8 | 2.00 | Unknown |
|  | JK754995 | XP_003248042.1 | 2202$E6 | 2.13 | Unknown |
|  | GW917618 | XP_976015.1 | 2202B3 | 0.34 | Unknown |
|  | FK669293 | ADI61823.1 | 2202D12 | 4.45 | Unknown |
|  | FK669216 | XP_969170.1 | 2202F12 | 2.02 | Unknown |
|  | FK669133 | EFA10016.1 | 2301H6 | 0.50 | Unknown |
|  | JK754994 | EFA01018.1 | 2401$E6 | 2.03 | Unknown |
|  | JK754998 | XP_972121.1 | 2401$E9 | 2.16 | Unknown |
|  | FK669334 | XP_966951.1 | 2503A12 | 0.18 | Unknown |
|  | JK817578 | XP_001809216.1 | 2503H1 | 9.60 | Unknown |
|  | FK669299 | XP_970038.1 | 2602B1 | 2.52 | Unknown |
|  | GW917415 | XP_973270.1 | 2603F8 | 2.13 | Unknown |
|  | FK669239 | XP_973726.1 | 2701$E2 | 2.53 | Unknown |
|  | FK669061 | EFA12191.1 | 2701A6 | 2.76 | Unknown |
|  | JK754991 | XP_976386.1 | 2703D9 | 0.49 | Unknown |
|  | JK754969 | XP_970896.1 | 2704D8 | 0.44 | Unknown |
|  | JK754970 | XP_970253.1 | 2903D5 | 0.33 | Unknown |
|  | JK754974 | EFA13284.1 | 2904C4 | 0.44 | Unknown |
|  | FK669256 | XP_972864.1 | 3003A11 | 0.49 | Unknown |
|  | FK669425 | XP_967856.1 | 3004F12 | 2.19 | Unknown |
|  | JK755005 | ABF20545.1 | 3101D7 | 0.50 | Unknown |
|  | JK755001 | ZP_04923980.1 | 3101D8 | 0.42 | Unknown |
|  | JK754990 | EFA07175.1 | 3102C11 | 0.39 | Unknown |
|  | FK668898 | NP_001103905.1 | 3103B10 | 0.42 | Unknown |
|  | JK754971 | XP_968291.1 | 3103F7 | 0.45 | Unknown |
|  | FK669445 | XP_967856.1 | 3201D12 | 2.12 | Unknown |
|  | JK755004 | XP_968747.2 | 3202C10 | 0.34 | Unknown |
|  | FK669400 | EFA02557.1 | 3301H4 | 0.25 | Unknown |
|  | FK669467 | XP_976415.1 | 3303A11 | 0.43 | Unknown |
|  | FK668988 | EEZ97810.1 | 3304D4 | 0.32 | Unknown |
|  | JK754978 | ABI35978.1 | 3304F1 | 0.46 | Unknown |
|  | JK755014 | EFA03852.1 | 3401A8 | 0.45 | Unknown |
|  | FK669258 | XP_972864.1 | 3401B7 | 0.41 | Unknown |
|  | FK669257 | XP_972251.1 | 3402C8 | 0.25 | Unknown |
|  | JK754972 | XP_970253.1 | 3404B10 | 0.20 | Unknown |
|  | FK669612 | EFA03938.1 | 3404F1 | 2.24 | Unknown |
|  | FK669393 | EEZ97241.1 | 3602C8 | 0.34 | Unknown |
|  | FK669469 | EFA03860.1 | 3603$E2 | 0.42 | Unknown |
|  | JK754992 | XP_976414.2 | 3604A5 | 0.49 | Unknown |
|  | FK668903 | XP_968738.2 | 3703B3 | 0.31 | Unknown |
|  | JK754996 | XP_972339.2 | 3704$E5 | 2.06 | Unknown |
|  | FK669319 | XP_972914.1 | 3801F4 | 2.30 | Unknown |
|  | GW917389 | EFA13411.1 | 3803D6 | 0.36 | Unknown |
|  | JK754962 | XP_966526.1 | 3804C2 | 2.00 | Unknown |
|  | FK669049 | XP_972957.2 | 3902G1 | 0.49 | Unknown |
|  | FK669232 | EFA01365.1 | 3902G6 | 8.16 | Unknown |
|  | FK669385 | XP_001627617.1 | 4002D10 | 3.50 | Unknown |
|  | FK669123 | XP_966730.1 | 4003H6 | 2.41 | Unknown |
|  | JK754963 | XP_973647.1 | 4102H4 | 2.22 | Unknown |
|  | FK669237 | EEZ99235.1 | 4103$E7 | 2.36 | Unknown |
|  | JK755010 | EFA09940.1 | 4301F3 | 0.35 | Unknown |
|  | FK669115 | XP_976120.1 | 4304C9 | 2.61 | Unknown |
|  | JK754979 | XP_972987.1 | 4401A9 | 0.46 | Unknown |
|  | JK817579 | EFA12867.1 | 4402B5 | 0.40 | Unknown |
|  | FK669041 | EFA05977.1 | 4402D6 | 0.47 | Unknown |
|  | JK754977 | XP_973103.2 | 4403B11 | 0.36 | Unknown |
|  | JK754989 | XP_967284.2 | 4404$E7 | 2.19 | Unknown |
|  | FK669403 | XP_972251.1 | 4503G2 | 0.43 | Unknown |
|  | JK755011 | EFA13447.1 | 4504$E11 | 0.39 | Unknown |
|  | FK669125 | XP_967394.1 | 4603F4 | 6.75 | Unknown |
|  | JK755009 | XP_974937.2 | 4701G10 | 0.47 | Unknown |
|  | JK754984 | EDN59046.1 | 4703A4 | 2.18 | Unknown |
|  | FK669042 | EFA01479.1 | 4703G1 | 0.46 | Unknown |
|  | JK755008 | XP_001809758.1 | 4801D8 | 0.49 | Unknown |
|  | JK754985 | XP_966630.2 | 4802A10 | 2.04 | Unknown |
|  | GW917218 | EFA12644.1 | 4804C5 | 2.12 | Unknown |
|  | JK755000 | EHN87095.1 | 4804D5 | 0.47 | Unknown |
|  | FK669197 | EFA02259.1 | 4901D1 | 0.20 | Unknown |
|  | FK668989 | EEZ97810.1 | 4902B9 | 0.49 | Unknown |
|  | JK754997 | ABK26259.1 | 5003D11 | 0.49 | Unknown |
|  | GW917390 | EHN84979.1 | 5003G7 | 0.33 | Unknown |
|  | JK754968 | XP_968291.1 | 5204B3 | 0.36 | Unknown |
|  | JK754993 | XP_003241325.1 | 5301C6 | 0.40 | Unknown |
| No match | | | | | |
|  | JK754873 |  | 0101$E1 | 2.78 | No match |
|  | FK669661 |  | 0101H4 | 0.38 | No match |
|  | JK754928 |  | 0102A8 | 2.33 | No match |
|  | GW917604 |  | 0102D11 | 0.16 | No match |
|  | JK754911 |  | 0102D6 | 2.13 | No match |
|  | FK669483 |  | 0103B1 | 0.44 | No match |
|  | FK669663 |  | 0103C12 | 2.19 | No match |
|  | GW917687 |  | 0103C2 | 0.33 | No match |
|  | GW917675 |  | 0104$E2 | 2.01 | No match |
|  | JK754891 |  | 0104C10 | 0.20 | No match |
|  | FK669840 |  | 0202C4 | 26.99 | No match |
|  | JK754933 |  | 0204$E4 | 2.50 | No match |
|  | JK754924 |  | 0301$E12 | 2.02 | No match |
|  | JK754944 |  | 0301C10 | 2.10 | No match |
|  | FK669295 |  | 0301H5 | 2.28 | No match |
|  | FK669711 |  | 0302B9 | 2.06 | No match |
|  | JK754900 |  | 0303A2 | 2.19 | No match |
|  | FK669536 |  | 0303H5 | 0.27 | No match |
|  | FK669096 |  | 0304F1 | 0.42 | No match |
|  | FK669626 |  | 0402$E6 | 2.85 | No match |
|  | JK754912 |  | 0403F6 | 0.36 | No match |
|  | GW917628 |  | 0404$E8 | 2.12 | No match |
|  | FK669539 |  | 0501G11 | 0.44 | No match |
|  | JK754948 |  | 0503F10 | 0.39 | No match |
|  | JK754899 |  | 0601G8 | 2.24 | No match |
|  | JK754949 |  | 0602$E3 | 0.50 | No match |
|  | FK669540 |  | 0603H9 | 0.41 | No match |
|  | FK669628 |  | 0604G5 | 2.96 | No match |
|  | JK754917 |  | 0701G2 | 0.37 | No match |
|  | JK754942 |  | 0704$E3 | 2.14 | No match |
|  | JK754920 |  | 0802B2 | 0.48 | No match |
|  | GW917543 |  | 0803G7 | 2.14 | No match |
|  | JK754888 |  | 0804H6 | 2.26 | No match |
|  | GW917544 |  | 0901$E4 | 2.24 | No match |
|  | GW917721 |  | 0901D7 | 0.32 | No match |
|  | FK669632 |  | 0902$E9 | 2.24 | No match |
|  | JK754902 |  | 0904$E4 | 2.59 | No match |
|  | JK754898 |  | 1004C10 | 2.03 | No match |
|  | JK754904 |  | 1101$E3 | 2.04 | No match |
|  | JK754951 |  | 1102B10 | 0.47 | No match |
|  | JK754950 |  | 1102F12 | 0.48 | No match |
|  | FK669625 |  | 1102G11 | 2.01 | No match |
|  | JK754884 |  | 1201$E9 | 2.04 | No match |
|  | JK754955 |  | 1201H9 | 0.29 | No match |
|  | JK754869 |  | 1203H12 | 2.04 | No match |
|  | FK669833 |  | 1204A10 | 43.84 | No match |
|  | GW917562 |  | 1302$E8 | 2.28 | No match |
|  | FK669725 |  | 1302F11 | 2.64 | No match |
|  | GW91756 |  | 1303$E1 | 2.52 | No match |
|  | JK754937 |  | 1303G1 | 0.42 | No match |
|  | JK754882 |  | 1404$E2 | 2.05 | No match |
|  | GW917569 |  | 1404C11 | 2.33 | No match |
|  | FK669730 |  | 1404G6 | 5.69 | No match |
|  | FK669732 |  | 1501D11 | 9.48 | No match |
|  | FK669733 |  | 1502$E6 | 2.17 | No match |
|  | JK754871 |  | 1502B7 | 2.77 | No match |
|  | GW917191 |  | 1503H8 | 2.02 | No match |
|  | GW917723 |  | 1504B2 | 0.34 | No match |
|  | JK754926 |  | 1602$E9 | 2.52 | No match |
|  | JK754954 |  | 1603$E6 | 0.38 | No match |
|  | GW917453 |  | 1603F1 | 2.21 | No match |
|  | FK669127 |  | 1603H12 | 2.29 | No match |
|  | GW917460 |  | 1604$E10 | 2.20 | No match |
|  | GW917461 |  | 1702$E4 | 2.37 | No match |
|  | GW917467 |  | 1702G8 | 66.38 | No match |
|  | JK754923 |  | 1703G7 | 0.49 | No match |
|  | FK669494 |  | 1703H6 | 0.33 | No match |
|  | JK754916 |  | 1704$E10 | 2.14 | No match |
|  | FK669620 |  | 1802H9 | 2.67 | No match |
|  | JK754947 |  | 1803H3 | 0.43 | No match |
|  | JK754931 |  | 1804A1 | 2.24 | No match |
|  | GW917408 |  | 1804H11 | 0.37 | No match |
|  | GW917469 |  | 1901B4 | 2.32 | No match |
|  | FK669671 |  | 1901H10 | 0.39 | No match |
|  | JK754946 |  | 1903C4 | 2.08 | No match |
|  | JK754929 |  | 1904$E3 | 0.48 | No match |
|  | FK669387 |  | 1904D10 | 31.28 | No match |
|  | FK669419 |  | 2001F1 | 0.20 | No match |
|  | FK669637 |  | 2004G11 | 3.08 | No match |
|  | FK669832 |  | 2101C8 | 14.29 | No match |
|  | FK669496 |  | 2101G12 | 0.33 | No match |
|  | JK754895 |  | 2102A8 | 2.01 | No match |
|  | FK669633 |  | 2103B6 | 3.03 | No match |
|  | JK754922 |  | 2104B6 | 2.03 | No match |
|  | FK669466 |  | 2201A12 | 2.03 | No match |
|  | FK669746 |  | 2201B12 | 2.37 | No match |
|  | FK669696 |  | 2201B2 | 2.14 | No match |
|  | JK754907 |  | 2201B7 | 0.45 | No match |
|  | JK754930 |  | 2201H6 | 0.35 | No match |
|  | JK754925 |  | 2203G1 | 0.48 | No match |
|  | FK669635 |  | 2204$E5 | 34.84 | No match |
|  | JK754892 |  | 2301A4 | 2.32 | No match |
|  | JK754894 |  | 2302A6 | 2.06 | No match |
|  | FK669841 |  | 2303A11 | 27.85 | No match |
|  | FK669750 |  | 2303B10 | 11.13 | No match |
|  | JK754939 |  | 2303G8 | 0.49 | No match |
|  | JK754903 |  | 2402$E9 | 2.28 | No match |
|  | JK754914 |  | 2404$E10 | 2.26 | No match |
|  | GW917482 |  | 2501A11 | 2.00 | No match |
|  | FK669500 |  | 2501B3 | 0.44 | No match |
|  | JK754883 |  | 2503A1 | 2.36 | No match |
|  | JK754879 |  | 2503C5 | 0.34 | No match |
|  | JK754906 |  | 2603A12 | 2.14 | No match |
|  | JK754919 |  | 2701B8 | 0.34 | No match |
|  | FK669659 |  | 2702$E3 | 0.45 | No match |
|  | JK754918 |  | 2702A10 | 2.07 | No match |
|  | FK669758 |  | 2704A5 | 2.79 | No match |
|  | FK669564 |  | 2801A12 | 0.23 | No match |
|  | JK754913 |  | 2801H11 | 0.42 | No match |
|  | FK669643 |  | 2802A10 | 2.31 | No match |
|  | GW917488 |  | 2802A8 | 2.11 | No match |
|  | FK669760 |  | 2802B12 | 2.43 | No match |
|  | FK669828 |  | 2802H6 | 0.37 | No match |
|  | FK669473 |  | 2804D3 | 0.49 | No match |
|  | JK754890 |  | 2902$E2 | 2.04 | No match |
|  | JK754875 |  | 2902H9 | 0.38 | No match |
|  | JK754921 |  | 3002B6 | 0.30 | No match |
|  | JK754893 |  | 3003H12 | 2.73 | No match |
|  | FK668934 |  | 3004B7 | 9.06 | No match |
|  | JK754880 |  | 3102A12 | 2.06 | No match |
|  | FK669304 |  | 3102G6 | 50.25 | No match |
|  | FK669641 |  | 3103$E10 | 3.14 | No match |
|  | FK669347 |  | 3103B8 | 11.93 | No match |
|  | FK668958 |  | 3104C2 | 0.40 | No match |
|  | GW917699 |  | 3201A10 | 2.27 | No match |
|  | FK669408 |  | 3203F9 | 0.31 | No match |
|  | FK669694 |  | 3301D5 | 0.47 | No match |
|  | FK669768 |  | 3302B5 | 2.05 | No match |
|  | FK669775 |  | 3402B1 | 5.04 | No match |
|  | JK754872 |  | 3402B2 | 2.01 | No match |
|  | JK754901 |  | 3402D4 | 0.47 | No match |
|  | JK754881 |  | 3504G5 | 2.29 | No match |
|  | FK669508 |  | 3601A3 | 0.46 | No match |
|  | FK669509 |  | 3602D10 | 0.50 | No match |
|  | FK669569 |  | 3603F6 | 0.43 | No match |
|  | FK669678 |  | 3701F11 | 0.48 | No match |
|  | JK754886 |  | 3703F10 | 2.10 | No match |
|  | FK669652 |  | 3703G5 | 0.25 | No match |
|  | FK669786 |  | 3704$E8 | 3.30 | No match |
|  | FK669787 |  | 3704C7 | 11.64 | No match |
|  | GW917579 |  | 3704F5 | 0.37 | No match |
|  | GW917580 |  | 3803C12 | 0.18 | No match |
|  | FK669836 |  | 3803F7 | 33.80 | No match |
|  | FK669571 |  | 3803G10 | 0.24 | No match |
|  | JK754885 |  | 3804C8 | 2.26 | No match |
|  | FK669303 |  | 3903A10 | 41.57 | No match |
|  | FK669680 |  | 3903B10 | 2.18 | No match |
|  | JK754910 |  | 3903H5 | 0.48 | No match |
|  | FK669471 |  | 3904A10 | 2.08 | No match |
|  | JK754905 |  | 3904B3 | 0.47 | No match |
|  | JK754887 |  | 3904H4 | 2.12 | No match |
|  | JK754934 |  | 4001$E10 | 2.13 | No match |
|  | FK669837 |  | 4001$E4 | 33.11 | No match |
|  | FK669640 |  | 4003C8 | 2.15 | No match |
|  | FK669465 |  | 4004A6 | 2.89 | No match |
|  | FK669796 |  | 4101F7 | 10.94 | No match |
|  | JK754935 |  | 4102B2 | 0.35 | No match |
|  | FK669618 |  | 4102C12 | 0.12 | No match |
|  | JK754878 |  | 4103F9 | 0.44 | No match |
|  | JK754943 |  | 4104B1 | 0.48 | No match |
|  | FK669627 |  | 4104F10 | 15.29 | No match |
|  | JK754877 |  | 4201F3 | 0.38 | No match |
|  | JK754909 |  | 4202$E7 | 2.02 | No match |
|  | FK669799 |  | 4202B1 | 2.77 | No match |
|  | JK754957 |  | 4204G3 | 0.50 | No match |
|  | FK669802 |  | 4301B11 | 2.82 | No match |
|  | JK754889 |  | 4301C8 | 2.01 | No match |
|  | FK669803 |  | 4301F7 | 10.82 | No match |
|  | JK754958 |  | 4303H10 | 0.47 | No match |
|  | JK754959 |  | 4304D7 | 0.41 | No match |
|  | JK754927 |  | 4304H10 | 0.47 | No match |
|  | FK669578 |  | 4401G11 | 0.19 | No match |
|  | FK669649 |  | 4402A3 | 2.25 | No match |
|  | FK669636 |  | 4403D9 | 2.22 | No match |
|  | GW917706 |  | 4404$E11 | 0.49 | No match |
|  | JK754941 |  | 4501H11 | 0.50 | No match |
|  | JK754952 |  | 4503C7 | 0.25 | No match |
|  | FK669806 |  | 4601B4 | 31.97 | No match |
|  | FK669619 |  | 4601D10 | 0.33 | No match |
|  | JK754953 |  | 4601H2 | 0.36 | No match |
|  | JK754956 |  | 4603F1 | 0.30 | No match |
|  | FK669809 |  | 4702D11 | 3.44 | No match |
|  | JK754940 |  | 4702D9 | 0.41 | No match |
|  | JK754896 |  | 4702G1 | 2.01 | No match |
|  | JK754938 |  | 4702G8 | 0.36 | No match |
|  | FK669842 |  | 4703B9 | 25.58 | No match |
|  | FK669581 |  | 4703F9 | 0.36 | No match |
|  | JK754945 |  | 4802F3 | 0.42 | No match |
|  | FK669516 |  | 4802G9 | 0.39 | No match |
|  | FK669517 |  | 4802H11 | 0.43 | No match |
|  | JK754936 |  | 4901H3 | 0.48 | No match |
|  | FK669521 |  | 4902F2 | 0.41 | No match |
|  | FK669522 |  | 4903F10 | 0.49 | No match |
|  | GW917708 |  | 4903G4 | 3.22 | No match |
|  | FK669683 |  | 5001H7 | 0.43 | No match |
|  | JK754870 |  | 5002D12 | 2.09 | No match |
|  | FK669703 |  | 5003A6 | 0.32 | No match |
|  | JK754908 |  | 5003C5 | 0.37 | No match |
|  | JK754874 |  | 5101G9 | 2.08 | No match |
|  | FK669611 |  | 5102B1 | 0.36 | No match |
|  | JK754932 |  | 5103F3 | 0.43 | No match |
|  | FK669685 |  | 5103F5 | 0.47 | No match |
|  | GW917534 |  | 5202A10 | 2.09 | No match |
|  | JK754915 |  | 5203F11 | 2.16 | No match |
|  | JK754897 |  | 5301G7 | 2.62 | No match |
|  | JK754876 |  | 5303A1 | 0.47 | No match |
| **^a^:** Bergapten-responsive genes are defined as having a two-fold or larger change (*P* ≤ 0.05) in gene expression compared to control. Induced genes are shaded red and repressed genes in green color.  **^b^:** The *E*-value cutoff is 10^-6^. | | | | | |
